# NPTX2 is a key component in the regulation of anxiety

**DOI:** 10.1038/s41386-018-0091-z

**Published:** 2018-05-11

**Authors:** Simon Chang, Philane Bok, Ching-Yen Tsai, Cheng-Pu Sun, Hsuan Liu, Jan M. Deussing, Guo-Jen Huang

**Affiliations:** 1 Department and Graduate Institute of Biomedical Sciences, College of Medicine, Chang Gung University, Taoyuan, Taiwan; 2 Department of Stress Neurobiology and Neurogenetics, Max Planck Institute of Psychiatry, Munich, Germany; 3 Institute of Molecular Biology, Academia Sinica, Taipei, Taiwan; 4 Institute of Biomedical Sciences, Academia Sinica, Taipei, Taiwan; 5 Molecular Medicine Research Center, Chang Gung University, Taoyuan, Taiwan; 6 Healthy Aging Research Center, Chang Gung University, Taoyuan, Taiwan; 7 Neuroscience Research Center, Chang Gung Memorial Hospital, Linkou, Taiwan

## Abstract

Anxiety disorders significantly impair quality of life. However, limited knowledge of the underlying mechanisms impedes the development of effective therapeutics. Previous studies have suggested that the expression of the *Nptx2* gene is associated with anxiety, but the neurobiological processes underlying this association remain unclear. We generated multiple mouse models with knockout or overexpression of *Nptx2* in specific brain regions and during different developmental stages to assess anxiety, adult neurogenesis, and glucocorticoid-related gene expression. Our results provide evidence that *Nptx2* expression in the adult hippocampus regulates anxiety in mice. Eliminating *Nptx2* expression in either the developing mouse brain or in adulthood leads to increased anxiety levels. The increase in anxiety was evident in hippocampus-specific *Nptx2* knockout mice, but not in an amygdala specific knockouts. Gene expression analysis revealed increased expression of glucocorticoid receptor target genes in *Nptx2* knockout mice after acute stress. Overexpression of *Nptx2* in the hippocampus alleviates stress-induced anxious behaviors and reverses the changes in expression of glucocorticoid receptor related genes. In conclusion, we demonstrate that *Nptx2* in the hippocampus performs a critical role in modulating anxiety, hippocampal cell proliferation, and glucocorticoid receptor related gene expression. Our results suggest *Nptx2* may be a potential target for anxiolytic therapeutics.

## Introduction

Anxiety disorders are highly prevalent and have the capacity to severely perturb quality of life. Unfortunately, the underlying molecular mechanisms that regulate normal and pathological mood states are currently poorly understood. We previously reported that *Nptx2* (also called NARP, neuronal activity-regulated pentraxin) is the most significantly upregulated transcript in the hippocampus in mice treated with either an antidepressant or exercise [1]. Other studies have shown that NPTX2 is altered in pathological states such as Alzheimer’s disease [2] and schizophrenia [3]. To date, not much is known about the role of NPTX2 in anxiety.

In situ hybridization (ISH) images from the Allen Brain Atlas (http://brain-map.org/) show that the gene is expressed in limbic and cortical regions. *Nptx2*, also named *Narp* (neuronal activity-regulated pentraxin) is a secreted protein, first identified in venom research [4]. NPTX2 protein is localized specifically to excitatory synapses in both pre- and postsynaptic compartments in the adult brain to induce the aggregation of neuronal α-amino-3-hydroxy-5-methyl-4-isoxazolepropionic acid receptors (AMPA receptors) [5]. NPTX2 interacts with other members of the pentraxin family, which include NPTX1 and NPTXR/3 to form complexes that bind to AMPA receptors [6, 7]. Evidence shows NPTX2 accumulates on parvalbumin-expressing interneurons to regulate homeostatic scaling of excitatory synapses [8]. Mice without NPTX2/NPTXR exhibit profound loss of GluA4 and AMPA receptor function in parvalbumin-expressing neurons, suggesting NPTXs plays an essential role in inhibition/excitation balance [9]. Interventions that influence emotional responses also regulate *Nptx2* expression. Long-term treatment with antidepressants, electroconvulsive seizure, or brain-derived neurotrophic factor (BDNF) infusion all improve emotional status, as well as increase the expression of *Nptx2* in the hippocampus [10–14].

In this study, we generated loxP-flanked *Npxt2* knockout (KO) mice to examine the role of *Nptx2* on anxiety. This manipulation enabled precise elimination of *Nptx2* expression in a spatially and/or temporally controlled manner. Our data suggest that hippocampal *Nptx2* plays a critical role in regulating anxiety, progenitor cell proliferation, and the expression of genes that underlie the glucocorticoid response. Thus, our study provides strong evidence that hippocampal *Nptx2* regulates anxiety and reveals it as a potential target for novel treatments for stress-related anxiety disorders.

## Materials and methods

### Animals

Experiments were performed on sex balanced cohorts of nine-week-old *Nptx2* KO and wild- type littermate mice. *Nptx2* floxed mice were generated from ES cells from IMPC (International Mouse Phenotyping Consortium). *Sox1*::*Cre* mice (Acc No. CDB0525K) were genotyped as described previously [15]*. CAG*::*Cre*ER^*T2*^ transgenic mice were purchased from Jax Lab (Stock number: 004682). Behavioral tests were conducted during the dark phase. Mice were housed in a 12:12 h light–dark cycle at 22 °C and humidity of 60–70%. Animals had ad libitum access to food and water. All protocols in this study were reviewed and approved by the Institutional Animal Care and Use Committee at Chang Gung University, Taiwan (IACUC: CGU15-063).

*Crh* (*Crh*::*Cre*; Ai9) and *Crhr1* (*Crhr1*::*Cre*; Ai9) reporter mice for colocalization studies were generated by breeding respective Cre drivers [16] to Ai9 (R26^CAG::loxP-STOP-loxP-tdTomato^, stock no: 007905) mice purchased from the Jackson Laboratory. Animal experiments were conducted in accordance with the Guide for the Care and Use of Laboratory Animals of the Government of Bavaria, Germany.

### Behavioral testing

To assess anxiety behavior, mice were subjected to the open field, elevated-O-maze, light–dark box, and novelty-suppressed feeding tests. The movement of animals was tracked using Ethovision software. There was a one day rest between each test. Mice were removed randomly to a transferring cage for testing, and stayed in a separated cage with bedding, food and water before being transferred back to the home cage. All mazes are made of acrylic; there is no bedding in the maze during the test.

#### Open field test

Animals were allowed to freely move for 5 min in an arena (radius = 45 cm, inner circle with radius = 30 cm).

#### Light dark box

At the start of the test, mice were put in the covered dark (black) box and permitted to move freely between the dark and the coverless light (white) box for 5 min.

#### Elevated-O-maze

The apparatus consisted of two open arms and two closed arms of equal size. The maze was a circle (radius = 55 cm) elevated 60 cm above the floor. The closed arms featured a 15 cm wall. Mice were placed in the elevated-O-maze for 5 min under dim light. The time spent in the open arms and the number of open arms entries were measured.

#### Novelty-suppressed feeding

Food was removed from the home cage for 24 h before testing. For testing, mice were placed in a brightly lit standard rat cage featuring food pellets placed in the four corners. The latency to first approach the food, and the time spent eating in the 7-min test period was recorded manually using a stopwatch.

#### Home cage food consumption test

To measure chronic food consumption, food pellets were removed for 6 h prior to being replaced. Food intake was then measured for 1 h. The test was conducted during the active period of mice.

### Immunohistochemistry and quantification

Dissected brains were fixed in 4% paraformaldehyde overnight, following dehydration in 25% sucrose in phosphate-buffered saline (PBS). All sections for NPTX2, DCX (doublecortin), Ki67, BrdU (5-bromo-2’-deoxyuridine), and c-Fos staining were sliced at a thickness of 40 µm. Brain sections were mounted on SuperFrost Plus slides (Thermo) and dried overnight. Slides were then incubated in 0.01 M citric acid buffer for 15 min at 95 °C, 3% H_2_O_2_ for 10 min, rinsed in PBS, and incubated overnight at room temperature in NPTX2 antibody (1:1000, Proteintech), DCX antibody (1:250, Santa Cruz), Ki67 antibody (1:1000, Vector Lab), BrdU antibody (1:250, Accurate), or c-Fos antibody (1:1000, Santa Cruz). Subsequently, we used a standard IgG ABC kit (Vector Lab) procedure according to the manufacturer’s instructions and incubated the slide for 5–10 min with a Sigma DAB tablet. Sections were then counterstained with cresyl violet and mounted in DPX.

For BrdU/NeuN double labeling, sections were incubated in 2 M HCl for 30 min at 37 °C, neutralized in boric acid (Sigma) for 15 min (pH 8.5) and washed three times in PBS before incubation with BrdU antibodies (1∶250, Accurate) and NeuN (1∶400 Millipore). Following three washes in PBS (5 min each), sections were incubated with the fluorescent secondary antibody (1∶250, Alex Fluor 488 and Texas Red, Invitrogen) for 2 h in 0.3% Triton/PBS with 2% of goat serum.

For NPTX2, NeuN, and GFAP immunofluorescence, sections were mounted on SuperFrost Plus slides and dried overnight. Then, slides were incubated in 0.01 M citric acid buffer for 15 min at 95 °C, rinsed in PBS and incubated overnight at room temperature in rabbit anti-NPTX2 antibody (1:1000, Proteintech), mouse anti-NeuN antibody (1:400, Millipore), mouse anti-GFAP antibody (1:1000, Sigma). Slides were then incubated with suitable fluorescence secondary antibody (1:250 Alexa 488 and Alexa 568, Invitrogen) and then washed with PBS. Slides were cover slipped with mounting medium (Fluoromount-G, SouthernBiotech) and left to either air dry or cooled to −20 °C for photo shooting.

For quantification, all slides were randomized and coded before quantitative analysis. Slides (half brain) were examined under a 20× objective. DCX, Ki67, and BrdU labeled cells were counted on every eighth section through the entire rostrocaudal extent of the granule cell layer (six sections per animal). The number of cells counted was then multiplied by sixteen to obtain an estimate of the total number of DCX, Ki67, and BrdU-positive cells in the dentate gyrus. For c-Fos and NPTX2 labeled cells, we counted cells on every eighth section through the entire rostrocaudal extent of the dentate gyrus, CA1 and CA3 areas (six sections per animal). The raw data was then modified as detailed above.

### Double in situ hybridization

Mice (10-week old) were sacrificed by an overdose of isoflurane. Brains were removed and shock-frozen on dry ice. Frozen brains were cut into 20-μm thick sections and mounted on SuperFrost Plus slides. All sections were processed for double in situ hybridization using a protocol described in a previous study [17]. The following riboprobes were used: *Gad67*: 984–1940 bp of NM_008077; *Gad65*: 753–1600 bp of NM_008078; *Vglut1* (*Slc17a7*): 1716–2332 bp of NM_010484; *Nptx2*: 1668–2384 bp of NM_008078 and *Fkbp5*: 1481–2328 bp of NM_010220. These were generated from PCRII TOPO vectors containing the cDNA insert. Molecular cloning was done per the manufacturer’s instructions (TOPO® TA Cloning® Kit, Cat.450640, Thermo Fisher). Specific riboprobes were generated by PCR from T7 and SP6 primers using plasmids containing the above-mentioned cDNAs as templates. Antisense and sense cRNA probes were synthesized and labeled with S^35^UTP or dioxygenin (DIG) by in vitro transcription from 1.5 µg of respective PCR product used as templates. For DIG detection, anti-DIG-POD (Fab) antibody was used at a concentration of 1/400. Tiramide-biotin signal amplification (TSA) was performed using the NEL700A Kit as per the manufacturer’s instructions. Hybridized slides were dipped in autoradiographic emulsion (type NTB2) and developed after 1 week.

### Corticosterone assay

Blood samples were collected 0, 30, and 90 min after stress via facial vein puncture. Plasma was separated from whole blood by centrifugation and stored at −80 °C until used. For analysis, plasma was diluted 1:30 in buffer and measured using the Corticosterone EIA kit (Enzo Life Sciences) as per the manufacturer’s instructions.

### Viral vector preparation and injections

For the *Nptx2* knockdown experiment, an AAV9-Cre vector (AV-9-PV2004) and AAV9-eGFP control vector (AV-9-PV0101) were purchased from vector core, University of Pennsylvania. For *Nptx2* overexpression and control vectors, DNA fragments that encoded NPTX2 or eGFP were created by PCR and subcloned into the NotI site of a AAV9 virus construct. Recombinant AAV9 vectors were produced by a standard triple-plasmid transfection method and purified by two rounds of CsCl centrifugation. The physical vector titers of AAVs were quantified using a real-time PCR method to measure the number of packaged vector genomes.

For the viral vector injections, surgery was performed under anesthesia. Mice received bilateral injections of viral vector into the hippocampus in a volume of 1.5 µl each injection side (4 × 10^12^ GC/ml for AAV9-Cre and control-eGFP viral vector; 5 × 10^13^ GC/ml for AAV9-*Nptx2* and control-eGFP) under stereotaxic guidance. For hippocampal injections, there were four injection sites in each mouse (dorsal hippocampus: AP −2.2 mm, ML ± 2.0 mm from bregma and DV −1.8 mm from dura; ventral hippocampus: AP −3.0 mm, ML ± 3.0 and DV −3.3 mm). For amygdala injections, there were two injection sites in each mouse (AP −1.75 mm, ML ±3.1, DV −4.0 mm).

### Statistical analysis

The mean ± SEM was determined for each group. Statistical analysis was performed using Graphpad Prism software. Data were analyzed via an analysis of variance (ANOVA) or *t*-test, as appropriate. Differences were considered significant when *p* < 0.05.

### Supplementary methods

For the detailed description of RNA seq-based gene expression analysis, mRNA quantification, Western blot analysis, see Supplementary Methods and Materials.

## Results

### Generation of nervous system-specific *Nptx2* KO mice

**Fig. 1:**
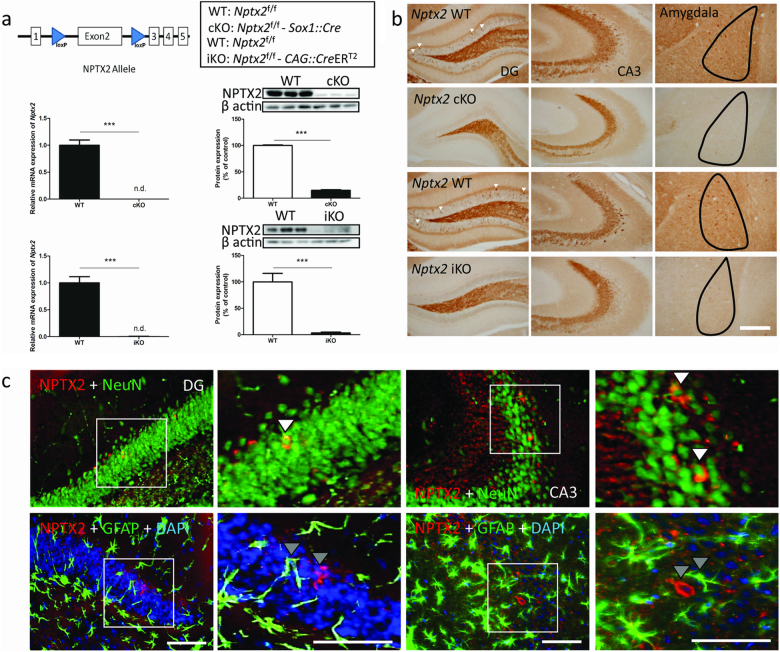
Generation of *Nptx2* conditional knockout mice. **a** *Nptx2* floxed mice were crossed with *Sox1*::*Cre* mice to knockout *Nptx2* specifically in the brain from early development or with *CAG*::*CreER*^*T2*^ to knockout *Nptx2* in adulthood. We confirmed the high knockout efficiency of *Nptx2* in the hippocampus at mRNA (*n* = 8) and protein levels (*n* = 6) for both models. **b** Immunohistochemistry confirmed the absence of NPTX2-positive cells in both the hippocampus and amygdala. **c** NPTX2 co-localized with NeuN (a marker for neurons), but not GFAP (a marker for glia). Gray arrowheads indicate no colocalization. White arrowheads indicate colocalization of two markers. Scale bar represents 200 µm. Values represent mean ± SEM. ***p *<* 0.001

To study the role of NPTX2 in emotion related behaviors, we confirmed the expression of *Nptx2* in the murine brain using ISH (Supplementary Fig. S1a–e). To develop nervous system-specific *Nptx2* conditional KO mice (cKO), we generated loxP-flanked *Nptx2* (*Nptx2*^f/f^) mice. We crossed *Nptx2* floxed mice with *Sox1*::*Cre* mice, which specifically express *Cre* throughout the neural tube from E11 [18]. These tissue specific cKO mice permitted an investigation into the functions of *Nptx2* in the brain. First, we demonstrated that *Nptx2* expression is dramatically decreased in the hippocampus by measuring mRNA via quantitative polymerase chain reaction (qPCR) (*p* *<* 0.0001, *t* *=* 10.14, *n* = 8) and protein levels via western blot (*p* < 0.0001, *t* = 50.81, *n* = 6) (Fig. 1a). Immunohistochemistry (IHC) showed only a few NPTX2-positive cells remained in cKO mice (Fig. 1b). On the IHC stains, we notice that some mossy fiber staining can be observed in *Nptx2* KO mice. Since the western and qPCR data demonstrates very low levels of this gene and protein in KO, this remaining staining should be a result of nonspecific staining. Next, by using IHC, we demonstrated that NPTX2 co-localizes with the neuronal marker NeuN, but not the glial marker GFAP (Fig. 1c). We then showed *Nptx2* mRNA co-localizes with *Vglut1* in the dentate gyrus (DG) and CA3 using double ISH. However, we detected minimal colocalization with *Gad65/Gad67*. This suggests that *Nptx2* is expressed primarily in excitatory neurons of the hippocampus (Supplementary Fig. S1f–i).

### Increased anxiety responses in *Nptx2* cKO mice

**Fig. 2:**
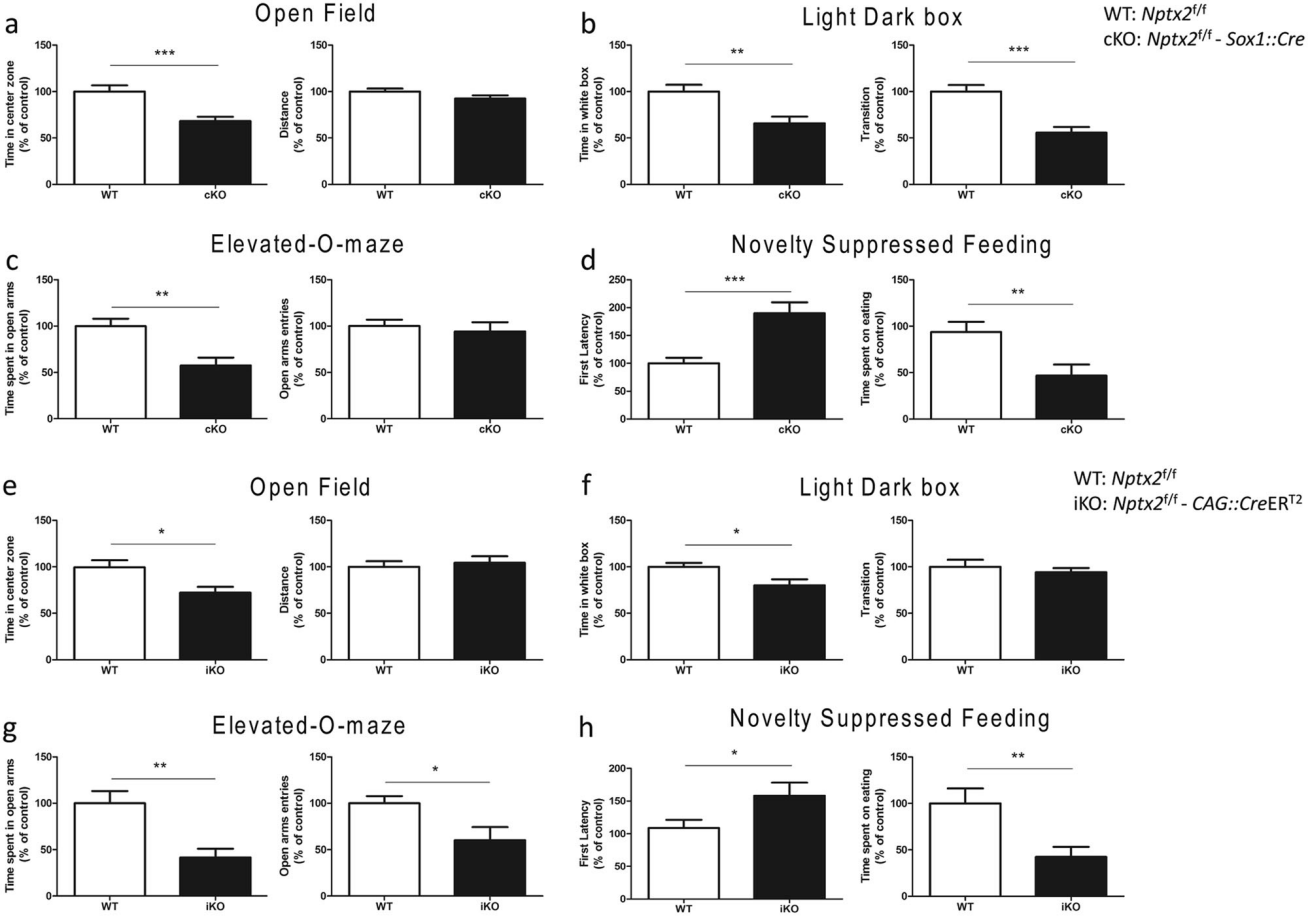
*Nptx2* knockout mice show increased anxiety. **a**–**d** Mice with brain-specific *Nptx2* KO (*Sox1*::*Cre*) (WT, *n* = 24; cKO, *n* = 21) and **e**–**h** tamoxifen-induced KO (*CAG*::*Cre*ER^*T*2^) (WT, *n* = 14; iKO, *n* = 11) showed increased anxiety behavior. **a**, **e** In the open field, KO mice spent less time in the central zone of the open field without a significant difference in distance traveled. **b**, **f** In the light–dark box, WT mice stayed longer in the white box and made more transitions between the two boxes. **c**, **g** In the elevated-O-maze, KO mice spent less time in the open arms and made a similar number of arm entries compared with WT. **d**, **h** In the novelty-suppressed feeding test, WT mice approached food pellets faster and spent more time eating than KO mice. Values represent mean ± SEM. *p *<* 0.05, **p *<* 0.01, ***p *<* 0.001

To investigate the relationship between *Nptx2* and anxiety, we subjected mice to a battery of behavioral tests (WT, *n* = 24; cKO, *n* = 21). This battery included the open field test, light–dark box, elevated-O-maze, and novelty-suppressed feeding. Overall, our results showed that *Nptx2* deletion robustly increased anxiety-like behaviors. In the open field, cKO mice spent less time in the center zone (*p* *<* 0.001, *t*_(43)_ = 3.84) with no significant difference in the total distance traveled (*p* *=* 0.11, *t*_(43)_ = 1.65) (Fig. 2a). In the light–dark box, cKO mice spent less time in the white box (*p* *=* 0.001, *t*_(43)_ = 3.33) and made fewer transitions between the two compartments (*p* *<* 0.0001, *t*_(43)_ = 4.64) (Fig. 2b). In the elevated-O-maze, we found that cKO mice spent less time in the open arms (*p* *=* 0.001, *t*_(43)_ = 3.65), but they did not exhibit a significant difference in the number of open arm entries (*p* *=* 0.63, *t*_(43)_ = 0.49) (Fig. 2c). Our results indicate cKO mice express higher levels of anxiety than WT mice. To further confirm this conclusion, we conducted an activity independent anxiety test, the novelty-suppressed feeding test. This test revealed that cKO mice exhibit a longer latency to approach food pellets (*p* *=* 0.0002, *t*_(43)_ = 4.18) and spend less time eating in a new environment (*p* *=* 0.008, *t*_(43)_ = 2.87) (Fig. 2d). WT and cKO mice had comparable home cage food consumption (*p* *=* 0.37, *t*_(43)_ = 1). Altogether, these results further demonstrate that *Nptx2* plays an important role in mediating anxiety.

### Altered hippocampal progenitor cell proliferation in *Nptx2* cKO mice

**Fig. 3:**
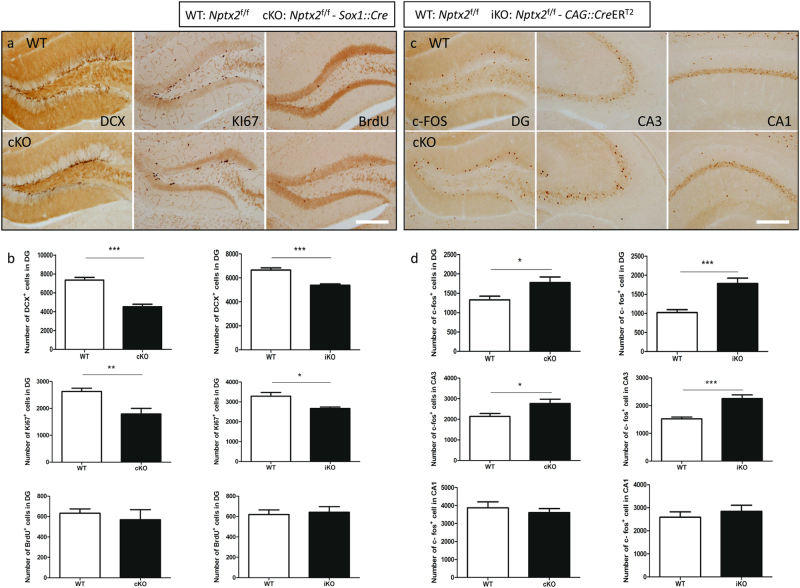
*Nptx2* knockout mice exhibit aberrant hippocampal cell proliferation and increased stress-induced neuronal activity. **a** Images of neurogenesis markers DCX, Ki67, and BrdU in the dentate gyrus of the hippocampus. **b** There were significantly reduced DCX and Ki67 labeled cell counts in the cKO hippocampus, but there were no significant differences in BrdU-positive cell numbers (left, *n* = 8). Inducible *Nptx2* KO had a similar decrease in cell proliferation (right, WT, *n* = 14; iKO, *n* = 11). **c**, **d** Images and quantification of c-Fos in the hippocampus DG, CA1, and CA3. Brain-specific KO (left, *n* = 8) and inducible KO mice (right, WT, *n* = 14; iKO, *n* = 11) have higher levels of c-Fos-positive cells in DG and CA3, but not in CA1 after acute restraint stress. Scale bar represents 200 µm. Values represent mean ± SEM. *p *<* 0.05, **p *<* 0.01, ***p *<* 0.001

*Nptx2* is expressed in the dentate gyrus and is upregulated following BDNF infusion, antidepressant treatment, and exercise [1, 12]. Since these factors play a significant role in neurogenesis [19, 20], we asked whether *Nptx2* contributes to the regulation of adult hippocampal neurogenesis. To investigate this, we injected mice with 5-bromo-2’-deoxyuridine (BrdU) (200 mg/kg, i.p.) to label newly born cells. Mice were killed 28 days after this BrdU injection to evaluate cell survival. We also assessed proliferation of neuronal progenitors by staining for doublecortin (DCX), a marker for immature neurons, and Ki67, a marker for cell proliferation. We demonstrated that cell proliferation and the number of immature neurons is dramatically decreased in *Nptx2* cKO mice (*n* = 8) as measured by Ki67 (*p* *=* 0.002, *t*_(14)_ = 3.75) and DCX (*p* *<* 0.0001, *t*_(14)_ = 6.98) staining. However, there was no significant difference in BrdU labeled cell counts (*p* *=* 0.5) (Fig. 3a, b left). Approximately 65% of the BrdU-positive cells were also positive for the neuronal marker, NeuN; however, there were no differences in the percentage of BrdU/NeuN co-labeling between the two groups (*n* = 6, *p* *=* 0.58, *t* = 0.68). These data reveal that despite the reduction of proliferating cells in the DG in cKO mice, neurogenesis is maintained, presumably due to a higher survival rate. This may reflect a compensation effect regulated by other factors to maintain adequate overall neurogenesis. Overall, these findings suggest that mice without *Nptx2* exhibit decreased progenitor cell proliferation but not altered neurogenesis.

### Altered stress-induced neuronal activity in *Nptx2* cKO mice

Altered plasma cortisol and stress-induced dysfunction through the hypothalamic–pituitary–adrenal (HPA) axis are important features in anxiety disorders [21]. To further investigate the role of *Nptx2* in response to stress, we measured plasma corticosterone levels at basal conditions and after 30 and 90 min after 30 min restraint stress. We found that corticosterone levels are higher in *Nptx2* cKO mice (*n* = 7) at the 30-min time point (*p* *=* 0.007, *t*_(12)_ = 3.23), but there were no significant differences at either the basal (*p* *=* 0.24, *t*_(12)_ = 1.24) nor 90-min time point (*p* *=* 0.19, *t*_(12)_ = 1.36) (Supplementary Fig. S2a). These results demonstrate that *Nptx2* cKO mice exhibit a greater HPA response to stress.

We then predicted that our observed differences in stress responsivity would manifest in concomitant neural activity measures in the hippocampus. c-Fos, an immediate early gene, is commonly used as a neuronal activity marker following a stimulus [22]. To characterize neuronal activity in WT and cKO hippocampi in response to stress (*n* = 8), we measured neuronal activation by quantifying the number of c-Fos-positive cells 90 min after 30 min of restraint stress in both WT and cKO mice. Interestingly, cKO mice showed more c-Fos-positive cells in the DG (*p* *=* 0.018, *t*_(14)_ = 2.68) and CA3 (*p* *=* 0.021, *t*_(14)_ = 2.61), but not in CA1 (*p* *=* 0.5, *t*_(14)_ = 0.68) and amygdala (*p* *=* 0.18, *t*_(14)_ = 1.41) (Fig. 3c, d left, Supplementary Fig. S3), suggesting *Nptx2* null mice display increased hippocampal neuronal activity in response to stress.

### The increased anxiety responses and altered hippocampal cell proliferation in *Nptx2* KO mice are not due to abnormal brain development

To determine whether the abnormal behaviors we observed in *Nptx2* cKO mice was a consequence of *Nptx2* inactivation during early embryonic development, we crossed *Nptx2* floxed mice with *CAG*::*Cre*ER^T2^ mice to induce loss of *Nptx2* specifically in adulthood upon tamoxifen administration (WT: *Nptx2*^f/f^; iKO: *Nptx2*^f/f^– *CAG*::*Cre*ER^T2^). Fourteen days after three daily injections of tamoxifen (100 mg/kg, i.p.), we assayed all mice using our behavioral battery described above (WT, *n* = 14; iKO, *n* = 11). In the open field, *Nptx2* iKO mice spent less time in the center zone (*p* *=* 0.02, *t*_(23)_ = 2.47) with no significant difference in the total distance traveled (*p* *=* 0.64, *t*_(23)_ = 0.46) (Fig. 2e). In the light–dark box test, iKO mice spent less time in the light compartment (*p* *=* 0.012, *t*_(23)_ = 2.7) with no significant differences in the number of transitions between the two boxes (*p* *=* 0.54, *t*_(23)_ = 0.61) (Fig. 2f). In the elevated-O-maze, iKO mice spent less time in the open arms (*p* *=* 0.004, *t*_(23)_ = 3.5) and made fewer entries into the open arms (*p* *=* 0.023, *t*_(23)_ = 2.57) (Fig. 2g). Finally, iKO mice showed a longer latency to approach food pellets (*p* *=* 0.011, *t*_(23)_ = 2.79) and spent less time eating (*p* *=* 0.01, *t*_(23)_ = 2.89) (Fig. 2h) in the novelty-suppressed feeding test. We also found no significant differences in home cage food consumption between WT and iKO mice (*p* *=* 0.15, *t*_(23)_ = 2.3). We assayed both *Nptx2* mRNA and protein levels in these mice (qPCR: *p* *=* 0.0002, *t* = 13.28, *n* = 8; Western blot: *p* *=* 0.0001, *t* = 4.81, *n* = 6). IHC analysis confirms there were significantly fewer NPTX2-positive cells in the hippocampus and amygdala in *Nptx2* iKO after tamoxifen injection (Fig. 1a, b).

We further analyzed hippocampal neurogenesis in these *Nptx2* inducible KO mice (WT, *n* = 14; iKO, *n* = 11). We found mice with induced *Nptx2* deletion display exhibit fewer DCX (*p* *<* 0.0001, *t*_(23)_ = 5.39) and Ki67 (*p* *=* 0.01, *t*_(23)_ = 2.8) positive cells in the DG, but no significant differences in BrdU-positive cells (*p* *=* 0.76, *t*_(23)_ = 0.31) (Fig. 3b right). However, induced *Nptx2* deletion yielded no significant differences in the plasma corticosterone level at any time after restraint stress (WT, *n* = 14; iKO, *n* = 11) (Supplementary Fig. S4b). Inducible KO mice exhibited greater c-Fos-positive cells in the DG (*p* *<* 0.0001, *t*_(23)_ = 5.06) and CA3 (*p* *<* 0.0001, *t*_(23)_ = 5.21), but not CA1 (*p* *=* 0.5, *t*_(23)_ = 0.69) and amygdala (*p* *=* 0.085, *t*_(23)_ = 1.81), 90 min following 30 min of restraint stress (Fig. 3d right and Supplementary Fig. S3). These data suggest that the altered anxiety and progenitor cell proliferation can occur as a consequence of *Nptx2* inactivation in adulthood, and not only because of developmental defects. Furthermore, we demonstrate that these changes in anxiety when the gene is deleted in adulthood are not due to corticosterone overproduction after stress.

### Hippocampus-specific knockout of *Nptx2* is sufficient to increase anxiety responses

**Fig. 4:**
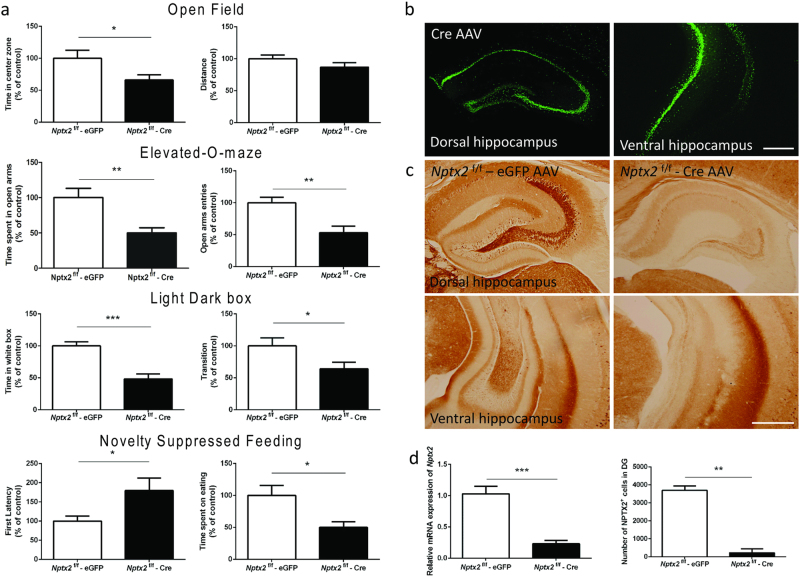
Hippocampal *Nptx2* knockdown by AAV-Cre injection causes increased anxiety. **a** Mice injected with AAV-*Cre* virus featured significantly increased anxiety. This is demonstrated by a decrease in time spent in the central zone (open field), less time spent in the open arms and fewer entries to the open arms (elevated-O-maze), spent less time in and made less transition to the white box (light–dark box), exhibited a greater latency to approach food pellets and spent less time eating (novelty-suppressed feeding test) compared to mice with AAV-eGFP virus injection (*n* = 10). **b**, **c** Viral vector expression (eGFP) and antibody staining of NPTX2 in the dorsal and ventral hippocampus. Immunohistochemistry confirmed there were no NPTX2-positive cells in the hippocampus. **d** mRNA expression of *Nptx2* and NPTX2-positive cell number in the hippocampus (*n* = 6). Scale bar represents 500 µm. Values represent mean ± SEM. *p *<* 0.05, **p *<* 0.01, ***p *<* 0.001

Since chronic antidepressant treatment or exercise upregulates *Nptx2* expression in the hippocampus [1, 13] and *Nptx2*-deficient mice display more c-Fos-positive cells in the DG and CA3 regions but not the amygdala after acute stress, we hypothesized that hippocampal *Nptx2* modulates anxiety behavior. To answer this, we injected either AAV-*Cre* or AAV-eGFP viral vectors into both the dorsal and ventral hippocampus in *Nptx2*^f/f^ mice (*n* = 10). Fourteen days after surgery, we assayed mice in our behavioral battery: open field, light–dark box, elevated-O-maze, and novelty-suppressed feeding tests. Our results show that *Nptx2*^f/f^ mice injected with AAV-*Cre* (*Nptx2*^f/f^-*Cre*) showed increased anxiety compared to the injected control AAV-eGFP group (open field: distance, *p* *=* 0.17, *t*_(18)_ = 1.4; time in center zone, *p* *=* 0.04, *t*_(18)_ = 2.21; light–dark box: time in white box, *p* *<* 0.0001, *t*_(18)_ = 5.06; transitions, *p* *=* 0.03, *t*_(18)_ = 2.24; elevated-O-maze: time in open arms, *p* *=* 0.005, *t*_(18)_ = 3.31; open arms entries, *p* *=* 0.003, *t*_(18)_ = 3.45; novelty-suppressed feeding: first latency, *p* *=* 0.03, *t*_(18)_ = 2.27; time spent on eating, *p* *=* 0.01, *t*_(18)_ = 2.77; food consumption, *p* *=* 0.32, *t*_(18)_ = 1.3) (Fig. 4a). These results are consistent with our data from cKO and iKO animal models.

Next, we subjected these mice to acute restraint stress and collected plasma after 30 minutes to assay corticosterone level. We found no significant differences in stress-induced plasma corticosterone levels between the two groups (*p* *=* 0.73, *t* = 0.35, *n* = 8) (Supplementary Fig. S2c). These results suggest that hippocampal *Nptx2* does not alter stress-induced corticosterone release. After harvesting the hippocampal tissue, we confirmed local inactivation of hippocampal *Nptx2* in this cohort of mice measured by two assays (qPCR, *p* *=* 0.0003, *t* = 6.19; IHC, *p* *=* 0.008, *t* = 10.58, *n* = 6) (Fig. 4b–d).

The amygdala plays a crucial role in fear and anxiety [23]. Since *Nptx2* is expressed in amygdala, we examined the contribution of amygdala *Nptx2* in anxiety via injecting AAV-*Cre* or AAV-eGFP viral vectors into the amygdala in *Nptx2*^f/f^ mice (*n* = 8). Fourteen days after surgery, we assayed mice in the behavioral battery described above. Our results showed no significant differences between amygdala *Nptx2* knockout and controls in the open field test (distance *p* *=* 0.49, *t*_(14)_ = 0.7; time in center zone *p* *=* 0.47, *t*_(14)_ = 0.75), light–dark box (time in white box *p* *=* 0.63, *t*_(14)_ = 0.5; transition *p* *=* 0.21, *t*_(14)_ = 1.3), elevated-O-maze (time in open arms *p* *=* 0.46, *t*_(14)_ = 0.77; open arms entries *p* *=* 0.45, *t*_(14)_ = 0.77). For the novelty-suppressed feeding test, the amygdala *Nptx2* knockout mice spent more time eating, but we found no significant differences in first latency and food consumption (first latency *p* *=* 0.65, *t*_(14)_ = 0.47; time spent eating *p* *=* 0.0001, *t*(14) = 5.2; food consumption *p* *=* 0.52, *t*_(14)_ = 0.65) (Supplementary Fig. S4d–g). We confirmed local inactivation of amygdala *Nptx2* in these mice as measured by IHC (*p* *<* 0.0001, *t*_(14)_ = 7.25) (Supplementary Figure S4a–c). Altogether, these data suggest that *Nptx2* expression in the hippocampus, but not the amygdala, plays a critical role in anxiety-related behaviors.

### *Nptx2* modulates hippocampal corticotropin-releasing hormone and glucocorticoid-related pathways

To determine the mechanism through which *Nptx2* alters anxiety, we assessed differences in hippocampal gene expression between brain-specific *Nptx2* cKO and WT mice by RNA-sequencing (RNAseq; *n* = 3). We found 397 genes with differential expression. Of these, we selected 53 genes (*p* < 0.05, fold change > 1.5 or <−1.5), excluding genes with low expression (expression value > 0.1) (Supplementary Fig. S5). Among these candidate genes, we found that corticotropin-releasing hormone (*Crh*) was strongly upregulated (*p* *=* 0.00038, fold change = 1.69).

**Fig. 5:**
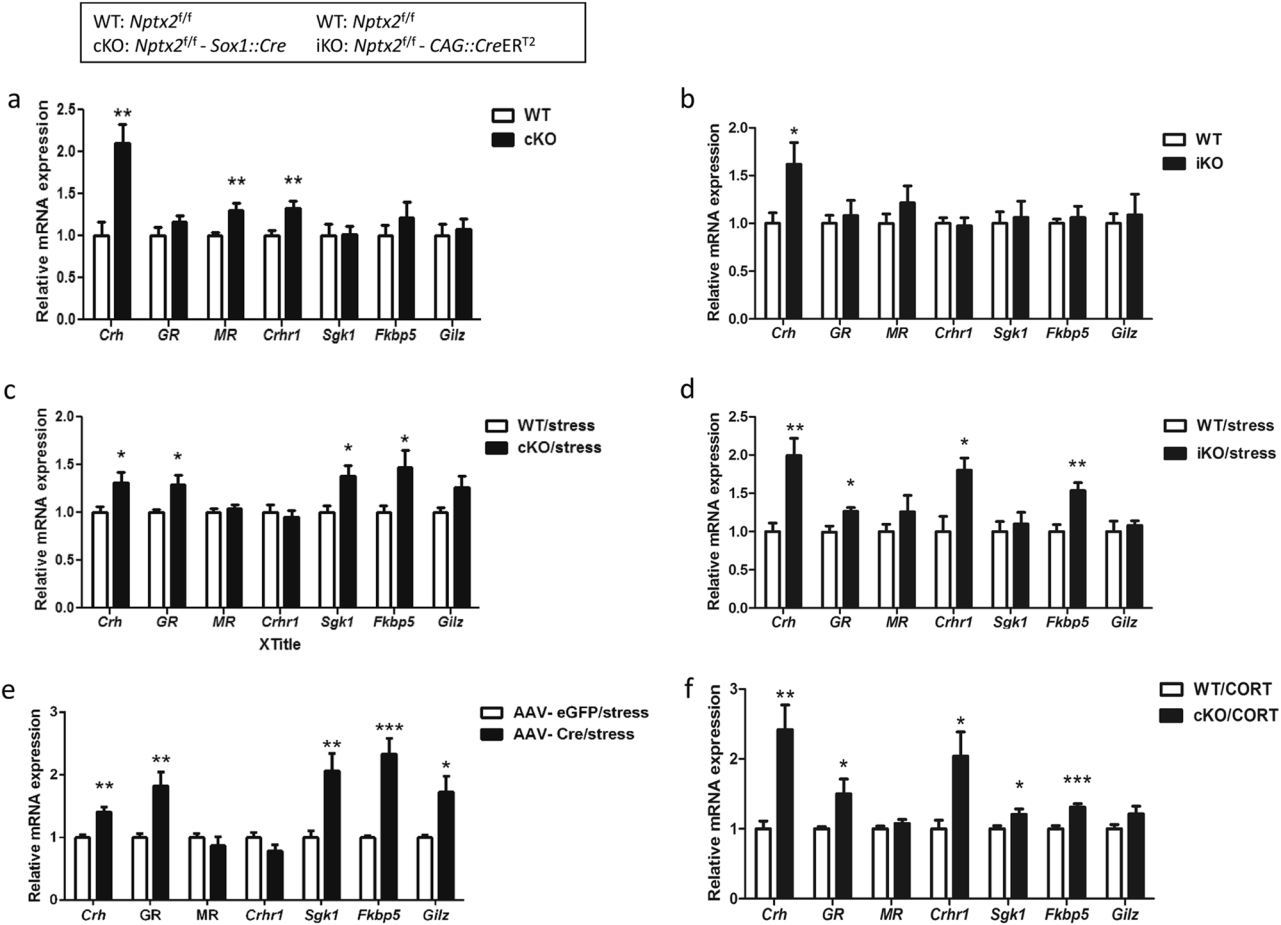
*Nptx2* regulates corticotrophin releasing hormone (*Crh*) and glucocorticoid pathway gene expression. **a**, **b** Expression of glucocorticoid-related genes (*Crh*, GR, MR, *Crhr1*, *Sgk1*, *Fkbp5*, and *Gilz*) in the hippocampus of *Nptx2* KO mice (brain-specific and tamoxifen-induced) (*n* = 8). **c**, **d** Gene expression in the hippocampus after 90 min of initiating 30 min of restraint stress in brain-specific KO or tamoxifen-induced KO mice (*n* = 8). **e** Gene expression in the hippocampus of control-eGFP and AAV-*Cre* injected mice after acute restraint stress (*n* = 6). **f** Gene expression of the hippocampus of WT and cKO mice 90 minutes after a single corticosterone injection (2.5 mg/kg, i.p. *n* = 8). Values represent mean ± SEM. *p *<* 0.05, **p *<* 0.01, ***p *<* 0.001

Hippocampal *Crh*, mineralcorticoid receptor (MR), glucocorticoid receptor (GR), and GR downstream genes play important roles in anxiety [24–27]. We examined whether *Nptx2* alters the expression of *Crh* and glucocorticoid-related genes by assaying the differences in hippocampal gene expression between *Nptx2* WT and cKO mice (*n* = 8). First, we confirmed increased expression of hippocampal *Crh* in *Nptx2* cKO mice (*p* *=* 0.001, *t*_(14)_ = 3.82) (Fig. 5a). Using IHC, we demonstrated that NPTX2 co-localizes with corticotropin-releasing hormone receptor 1 (CRHR1), but not CRH containing neurons (Supplementary Fig. S1l-o). Since *Nptx2* alters the expression of hippocampal *Crh*, we investigated the expression of glucocorticoid signaling related genes in the hippocampus, including *Nr3c1* (GR), *Nr3c2* (MR), *Crhr1*, *Sgk1*, *Fkbp5*, and *Gilz*. We found that *Nptx2* cKO mice exhibit higher levels of MR (*p* *=* 0.0018, *t*_(14)_ = 3.82) and *Crhr1* (*p* *=* 0.005, *t*_(14)_ = 3.36), but we found no significant differences in GR downstream genes (Fig. 5a). For tamoxifen-induced *Nptx2* deletion mice (*n* = 8), we confirmed upregulated *Crh* in *Nptx2* iKO mice (*p* *=* 0.02, *t*_(14)_ = 2.55). There were no differences in MR, *Crhr1*, and GR downstream gene expression (Fig. 5b).

Interactions between genetic predisposition and environment play an important role in the development of psychiatric disorders. To determine whether *Nptx2* alters the expression of glucocorticoid signaling related genes after stress, *Nptx2* WT and cKO mice (*n* = 8) experienced restraint stress for 30 min and were sacrificed after 90 min. We detected several gene expression changes in the hippocampus of cKO mice following acute stress, including upregulation of *Crh* (*p* *=* 0.03, *t*_(14)_ = 2.37), GR (*p* *=* 0.01, *t*_(14)_ = 2.89) and GR downstream genes, such as *Sgk1* (*p* *=* 0.01, *t*_(14)_ = 2.83), and *Fkbp5* (*p* *=* 0.02, *t*_(14)_ = 2.51) (Fig. 5c). We also detected similar changes in inducible *Nptx2* knockout mice (iKO; *Crh p* *=* 0.005, *t*_(14)_ = 3.68, GR *p* *=* 0.01, *t*_(14)_ = 3.01, *Crhr1 p* *=* 0.01, *t*_(14)_ = 3.17, *Fkbp5 p* *=* 0.003, *t*_(14)_ = 3.89) and *Nptx2* floxed mice injected with AAV-*Cre* into the hippocampus (*Nptx2*^f/f^ –*Cre*, *n* = 6; *Crh p* *=* 0.002, *t*_(14)_ = 4.47, GR *p* *=* 0.004, *t*_(14)_ = 3.68, *Sgk1 p* *=* 0.009, *t*_(14)_ = 3.31, *Fkbp5 p* *=* 0.0003, *t*_(14)_ = 5.32, *Gilz p* *=* 0.018, *t*_(14)_ = 2.83) (Fig. 5d, e). These results suggest *Nptx2* influences GR, *Sgk1*, and *Fkbp5* expression during stress.

Since stress increases plasma corticosterone, we wanted to determine whether the expression of these GR-related genes in *Nptx2*-deficient mice respond differently to elevated plasma corticosterone. We injected both WT and cKO mice (*n* = 8) with a single dose of corticosterone (CORT, 2.5 mg/kg, i.p.) to imitate the adrenal hormone stress response. We detected no significant differences in plasma corticosterone between WT and cKO mice 90 min after injection (WT 582.7 ± 44.71 ng/ml; KO 590.6 ± 56.94 ng/ml, *p* *=* 0.91, *t*_(14)_ = 0.1). Interestingly, cKO mice exhibited higher expression of *Crh* (*p* *=* 0.003, *t*_(14)_ = 3.6), GR (*p* *=* 0.04, *t*_(14)_ = 2.16), *Crhr1* (*p* *=* 0.019, *t*_(14)_ = 2.65), *Sgk1* (*p* *=* 0.04, *t*_(14)_ = 2.26) and *Fkbp5* (*p* *=* 0.0007, *t*_(14)_ = 4.54) (Fig. 5f). These results suggest that NPTX2 regulates sensitivity of GR signaling, rather than corticosterone levels per se.

### Overexpression of *Nptx2* in the hippocampus reduces stress-induced anxiety

To study the potential of *Nptx2* to be a target for novel treatments for anxiety, we overexpressed *Nptx2* in the hippocampus. C57BL/6 mice were separated into two groups (*n* = 10) and injected with either AAV-CB (chicken-beta actin)-*Nptx2* viral vector (OE) or with an AAV-CB-eGFP viral vector (Control) into the hippocampus. Fourteen days after surgery, we subjected all mice to behavioral testing. Our results showed no significant differences between OE mice and controls in the open field test, light–dark box, elevated-O-maze, novelty-suppressed feeding (Supplementary Fig. S6). Hippocampal tissues were harvested and we detected a robust increase in *Nptx2* mRNA expression in AAV-CB-*Nptx2* viral vector injecting group compared to eGFP control (*p* *=* 0.002, *t* = 3.79. *n* = 8). These results show that increased hippocampal *Nptx2* expression does not alter anxiety.

**Fig. 6:**
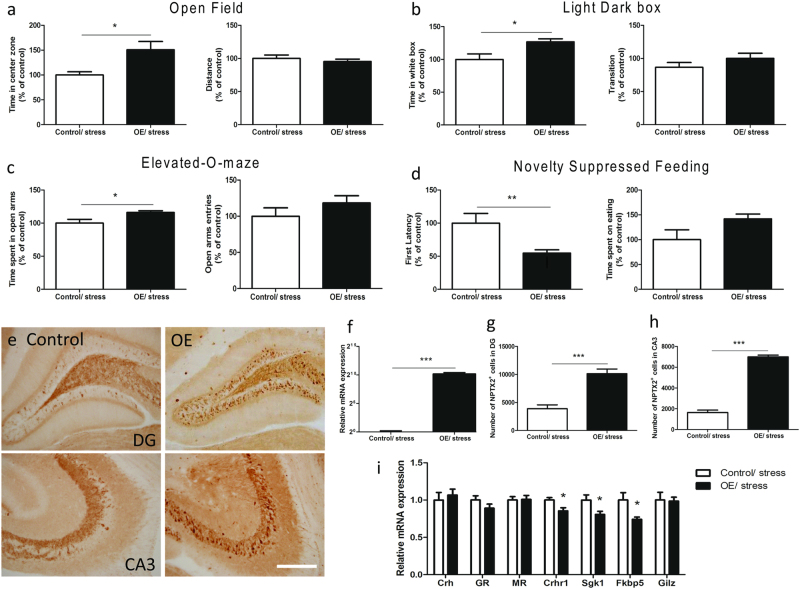
Overexpression of *Nptx2* alleviates stress-induced anxiety and reverses the expression of stress-related genes. **a**–**d** Hippocampal *Nptx2* overexpression alleviated stress-induced anxiety (*n* = 10). **e** Immunohistochemistry demonstrated an increase in NPTX2-positive cells in the hippocampus of *Nptx2* overexpression mice. **f** qPCR confirmed increased mRNA expression of *Nptx2* in the hippocampus (*n* = 7). **g**, **h** Cell numbers of NPTX2 in DG and CA3 measured by Immunohistochemistry (*n* = 6). **i** The expression of glucocorticoid-related genes in the hippocampus of control and *Nptx2* overexpression mice after acute stress (*n* = 7). Scale bar represents 200 µm. Values represent mean ± SEM. *p *<* 0.05, **p *<* 0.01, ***p *<* 0.001

Because C57BL/6 is one of the least anxious inbred mouse strains [28], we tested whether increased *Nptx2* can reduce anxiety following stress. Fourteen days after injection of overexpression or control viral vector into the hippocampus (C57BL/6 mice, *n* = 10), all mice were subjected to 3 h of restraint stress. We initiated behavioral tests following 6 h of post-stress resting in the home cage. We found that *Nptx2* overexpressing mice exhibit less anxious behavior in all four tests compared to eGFP control mice (open field: distance *p* *=* 0.47, *t*_(18)_ = 0.73, time in center zone *p* *=* 0.01, *t*_(18)_ = 2.83; light–dark box: time in white box *p* *=* 0.01, *t*_(18)_ = 2.83, transitions *p* *=* 0.22, *t*_(18)_ = 1.25; elevated-O-maze: time in open arms *p* *=* 0.0.02, *t*_(18)_ = 2.57; open arms entries *p* *=* 0.24, *t*_(18)_ = 1.2; novelty-suppressed feeding: first latency *p* *=* 0.008, *t*_(18)_ = 2.93; time spent on eating *p* *=* 0.07, *t*_(18)_ = 1.89) (Fig. 6a–d). This suggests increased *Nptx2* is protective against stress-induced anxiety.

We then sacrificed these mice 90 min following 30 min of restraint stress and assayed plasma corticosterone. We found no significant differences in plasma corticosterone levels between OE and control mice (*p* *=* 0.4, *t* = 0.86, *n* = 8) (Supplementary Fig. S2d). After measuring *Nptx2* expression levels, we confirmed that mice injected with AAV-CB-*Nptx2* viral vector expressed higher levels of *Nptx2* mRNA (*p* < 0.0001, *t* = 6.17; *n* = 7) (Fig. 6f). We also confirmed that *Nptx2* overexpressing mice had more NPTX2-positive cells (DG *p* *=* 0.0002, *t* = 5.86; CA3 *p* < 0.0001, *t* = 18, *n* = 6) (Fig. 6e, g, h). After stress, *Nptx2* overexpressing mice exhibited reduced expression of *Crhr1*, *Sgk1*, and *Fkbp5* (*Crhr1 p* *=* 0.02, *t* = 2.68, *Sgk1 p* *=* 0.03, *t* = 2.42, *Fkbp5 p* *=* 0.04, *t* = 2.3; *n* = 7) (Fig. 6i). Intriguingly, among these GR-related genes, *Fkbp5* expression coincides with *Nptx2* present (knockout and overexpression) under stress condition. Double ISH showed that *Nptx2* co-localized with *Fkbp5* in the DG and CA3 (Supplementary Fig. S1j, k). Overall, these results indicate that increased *Nptx2* reverses stress-induced behavioral changes and decreases GR downstream gene expression.

## Discussion

We utilized three gene deletion methods to examine the role of *Nptx2* in anxiety: central nervous system-specific, tamoxifen-induced, and hippocampus-specific deletion. Our experiments demonstrate that the effects of *Nptx2* deletion on anxiety-like behavior are hippocampus-specific and not developmentally related. In addition, mice with *Nptx2* deletion exhibit decreased hippocampal cell proliferation and increased stress-induced neuronal activity. Furthermore, changes in expression of GR-related genes are consistent with the behavioral results.

Our data show that brain-specific *Nptx2* KO mice exhibit increased anxiety. These results are consistent with a recent study that demonstrated double KO mice for *Nptx2* and *Nptxr* exhibit increased anxiety in both the open field and elevated-O-maze [9]. However, in that study, methodological issues may have influenced its conclusions. Constitutive *Nptx2*^−/−^/*Nptxr*^−/−^ double KO mice were used for behavioral tests, but the control mice were half WT and the other half were C57BL/6J mice ordered from Jax lab, non-littermate controls. These confounding environmental factors, including differences in maternal care and colony origin, can impact the assayed measures. In our study, we used littermates for all the experiments, including behavioral tests. Our work provides a multi-pronged approach to clearly delineate the role of Nptx2 in stress and anxiety.

From our previous study we found that the expression of hippocampal *Nptx2* is the most significantly upregulated transcript after chronic exercise or antidepressant treatment. Both interventions have been shown increase neurogenesis [1]. Other reports also show antidepressant [13], electroconvulsive seizure [11], or BDNF infusion [12] increases the expression of *Nptx2* in the hippocampus. These factors also influence neurogenesis and emotional responses, thus we hypothesize that *Nptx2* may play roles in neurogenesis or anxiety. In this study, we show *Nptx2* influences cell proliferation, but not neurogenesis. Considerable controversy remains in the literature as to the link between neurogenesis and anxiety. The data we present here suggests that the anxiety phenotype is independent of neurogenesis in *Nptx2* KO mice. It remains possible that some link between the proliferation and the anxiety phenotype exists. Our study does not address the causality between hippocampal cell proliferation and anxiety.

In this study, we found that *Nptx2* mediates *Crh* and GR-related gene expression changes. *Crh* expression is significantly increased in mice with *Nptx2* deletion. Previous study has demonstrated that *Crh* overexpressing mice exhibit an anxious behavioral state [27]. Conversely, mice lacking *Crhr1* show an impaired stress response and reduced anxiety [29]. We measured expression of GR and its downstream genes. However, we found no significant basal differences between KO and WT mice. One possibility is that the glucocorticoid-related genes we assayed are not activated in the absence of stress. The interaction between genes and environment play an important role in the pathogenesis of anxiety. In acutely stressed mice we detected increased expression levels of GR and its downstream genes, *Sgk1* and *Fkbp5*, in *Nptx2* deleted mice. Corticosterone is an adrenal hormone known to activate GR, particularly after stress. We find no difference in the plasma corticosterone levels after stress in our *Nptx2* models. Injection of equivalent stress levels of corticosterone causes greater changes in GR-related gene expression in the *Nptx2* deleted mice. In combination these data support a model whereby NPTX2 regulates the sensitivity to GR activation.

Additionally, we found that mice overexpressing *Nptx2* were less anxious in response to stress and showed a reversal of the stress-induced increases in GR, *Sgk1*, and *Fkbp5* expression. Psychiatric disorders, including anxiety and posttraumatic stress disorder, implicate *Fkbp5* as a critical gene [30–32]. Several lines of evidence suggest that stress or glucocorticoids increase the expression of *Fkbp5* [33, 34]. FKBP5, a co-chaperone of GR, binds to HSP90–GR complex to lower the binding affinity to cortisol, resulting in a decrease of the sensitivity of GR [30, 35]. In the present study mice without *Nptx2* exhibit higher levels of *Fkbp5* expression after stress or corticosterone injection. The increased response of *Fkbp5* occurs after acute stress in the absence of a difference in corticosterone, suggesting that *Nptx2* regulates sensitivity to corticosterone, rather than regulating corticosterone levels per se. With regards to how the changes in Fkbp5 link to the changes in anxiety, it is unclear whether the increased *Fkbp5* drives the altered anxiety, or whether the altered anxiety drives changes in *Fkbp5* expression. Similarly, hippocampal expression of *Sgk1* mRNA is increased in depressed patients and rodents following chronic stress [25]. These results provide molecular evidence that *Nptx2* regulates anxiety-related genes after stress. However, in this study the changes of gene expression we detect could result from, rather be the cause of the anxiety phenotype in these mutant mice. Therefore a specific mechanism by which *Nptx2* alters stress-induced behavior through *Sgk1* and/or *Fkbp5* remains undetermined.

In conclusion, our results indicate that hippocampal *Nptx2* plays a critical role in modulating anxiety, altering stress sensitivity, and influencing the expression of GR downstream genes. Consequently, we suggest that hippocampal *Nptx2* may be a novel target for anxiolytic therapeutics. Further understanding of the underlying cellular mechanisms will help the generation of new management strategies in the treatment of anxiety and stress-induced disorders.

## Electronic supplementary material

Supplementary information
