# Supplementary material for: NPTX2 is a key component in the regulation of anxiety
Source: Neuropsychopharmacology. 2018 May 11;43(9):1943–53. doi: 10.1038/s41386-018-0091-z (PMC6046040; doi:10.1038/s41386-018-0091-z)
Supplement: Supplementary file 1 — Supplementary information [file 41386_2018_91_MOESM1_ESM.docx]

**Supplementary information**

Title: **NPTX2 is a key component in the regulation of anxiety**

Simon Chang, Philane Bok, Ching-Yen Tsai, Cheng-Pu Sun, Hsuan Liu, Jan M. Deussing, Guo-Jen Huang

Contents

Supplementary Methods and Materials ----------------------------------------------------------- 2-3

Figures S1-S6 ----------------------------------------------------------------------------------------- 4-9

**RNA seq-based gene expression analysis**

RNA was isolated using TRIzol reagent. cDNA libraries were prepared based on the TruSeq® Stranded Total RNA Sample Preparation Guide (Illumina, Part # 15031048). Equal concentrations of each library were sequenced using a NextSeq 500 (Illumina) platform to create pair-end 75-bp reads. The sequences were assessed for quality and trimmed for primer-adaptor sequences by using the RNA-seq alignment tool from BaseSpace (Illumina), followed by alignment to the mouse reference genome (mm10). The aligned data was transferred to Partek Genomics Suite and assigned to transcribed genes annotated in GENCODE M8. Relative expression of each gene was represented by TPM (Transcripts Per Kilobase Million). Partek Genomics Suite and statistical package were used to perform statistical analysis, hierarchical clustering, differential expression analysis, and pathway enrichment. The RNA sequencing data is available on NCBI (PRJNA355191).

**mRNA quantification**

Hippocampal tissue was collected from WT and KO mice. mRNA was extracted using a RNeasy Lipid Tissue Kit (Qiagen). cDNA was then made using SuperScript III reverse transcriptase (Invitrogen). Experiments were performed in duplicate. Gene expression levels were then calculated by the ^ΔΔCt^ method and normalized against a GAPDH control. Primers used in this study are in the below table.

|  | Forward | Reserve |
| --- | --- | --- |
|  |  |  |
| *Nptx2* | CGGAGCTGGAAGATGAGAAG | GGAAGGGACACTTTGAATGC |
|  |  |  |
| *Crh* | AGCCCTTGAATTTCTTGCAG | GCGGGACTTCTGTTGAGATT |
|  |  |  |
| GR (Nr3c1) | ACGCCGACTTGTTTATCTGG | GAAAAGGACGCCAGACTCC |
|  |  |  |
| MR(Nr3c2) | CCGGTATTGGACTTGCTGTT | CAGCCTGAAGTTGGCTCTCT |
|  |  |  |
| *Crhr1* | CTGCCATCCGGAAGAGGT | CTCTGTGGCCTGAGGTTCA |
|  |  |  |
| *Sgk1* | CTGCTCGAAGCACCCTTACC | TCCTGAGGATGGGACATTTTCA |
|  |  |  |
| *Fkbp5* | AGCACACATCCCGTGTTCTA | TGCTGGGTTCTCTCCATTGT |
|  |  |  |
| *Gilz* | AACACCGAAATGTATCAGACCC | GTTTAACGGAAACCAAATCCCCT |
|  |  |  |
| *Gapdh* | TGACGTGCCGCCTGGAGAAAC | CCGGCATCGAAGGTGGAAGAG |
|  |  |  |

**Western blot analysis**

Protein extracts were obtained by lysing tissue in 1% SDS. Samples were homogenized and heated at 100 °C for 10 minutes. Proteins were then separated by gel electrophoresis and electro-transferred onto nitrocellulose membranes. Blots were incubated with primary antibody (NPTX2 1:10000, Proteintech) in TBST and 5% fat free milk overnight at 4 °C. Subsequently, blots were washed and probed with the respective horseradish peroxidase secondary antibody for 1 h at room temperature. Immunoreactivity bands were visualized using ECL detection reagent (GE Healthcare, RPN2106). Assessment of the band intensities were performed using ChemiDoc MP from Bio-Rad.

**Supplementary Figures**

**
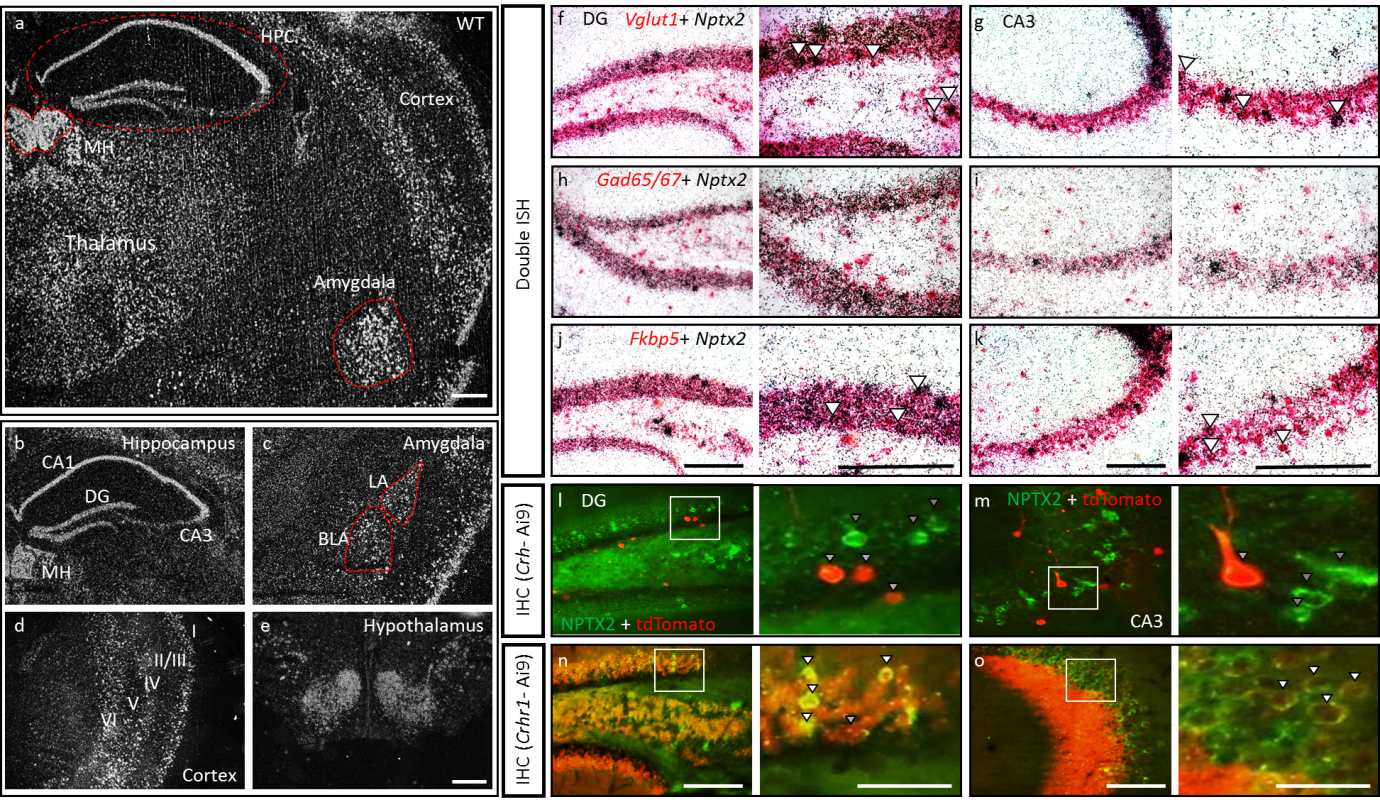
**

**Figure S1.** **Colocalization of *Nptx2* by double ISH or IHC**

(a-e) Expression of *Nptx2* mRNA in the mouse brain. (f and g) *Nptx2* and *Vglut1* co-localize in DG and CA3. White arrowheads indicate positive *Nptx2* signal expressed in glutamatergic (*Vglut1*) neurons. (h and i) No obvious *Nptx2* positive signal is expressed in GABAergic (*Gad65/67*) neurons in DG and CA3. (j and k) *Nptx2* and *Fkbp5* co-localize in DG and CA3. (l and m) NPTX2 is not expressed in CRH containing neurons in DG or CA3. (n and o) NPTX2 is co-localized with CRHR1 in DG and CA3. Grey arrowheads indicate no colocalization. White arrowheads indicate colocalization of two markers. Scale bar = 200 µm.

**
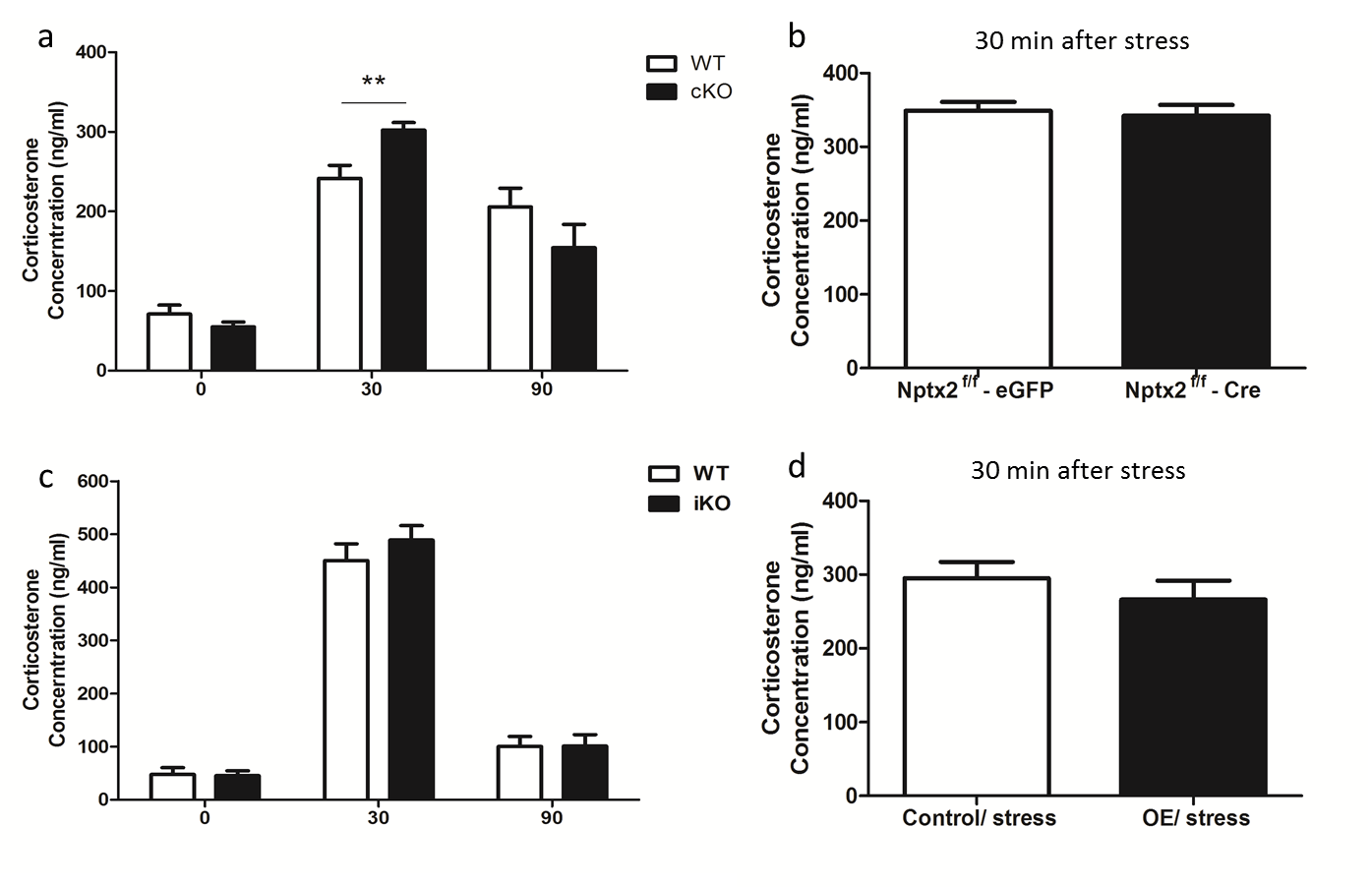
Figure S2. Plasma corticosterone after restraint stress**

(a and b) Plasma corticosterone at basal level, 30 minutes and 90 minutes after restraint stress. cKO mice have higher plasma corticosterone 30 minutes after stress (n = 7). In contrast, there were no significant differences in corticosterone concentration in iKO mice (n = 11). (c) There were no significant differences in plasma corticosterone 30 minutes after restraint stress between control and hippcampal *Cre*viral vector injected mice (n = 8). (d) No significant difference in plasma corticosterone between control and hippocampal *Nptx2*overexpressed mice 30 minutes after restraint stress (n = 8). Values represent mean ± SEM. ***p* < 0.01.

**
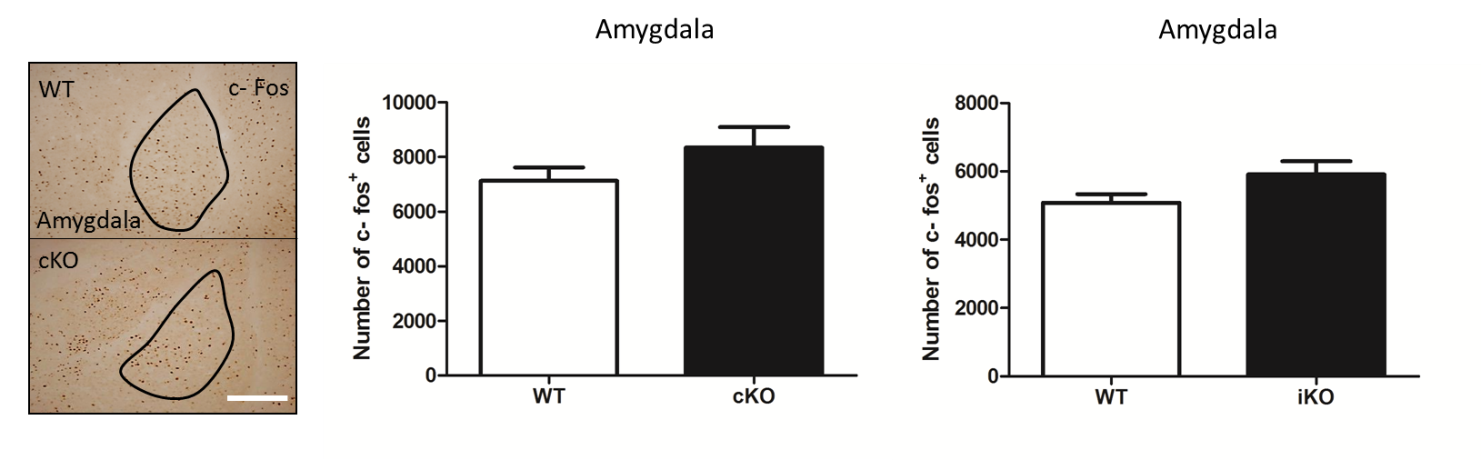
Figure S3. Stress-induced c-Fos positive cells number in *Nptx2* deficit mice**

There is no significant difference in c-Fos positive cell number in the amygdala 90 minutes following 30 minutes of restraint stress between WT and *Nptx2* deficient mice (cKO, n = 8; iKO, WT = 14, iKO = 11). Scale bar represents 200 µm. Values represent mean ± SEM.

**
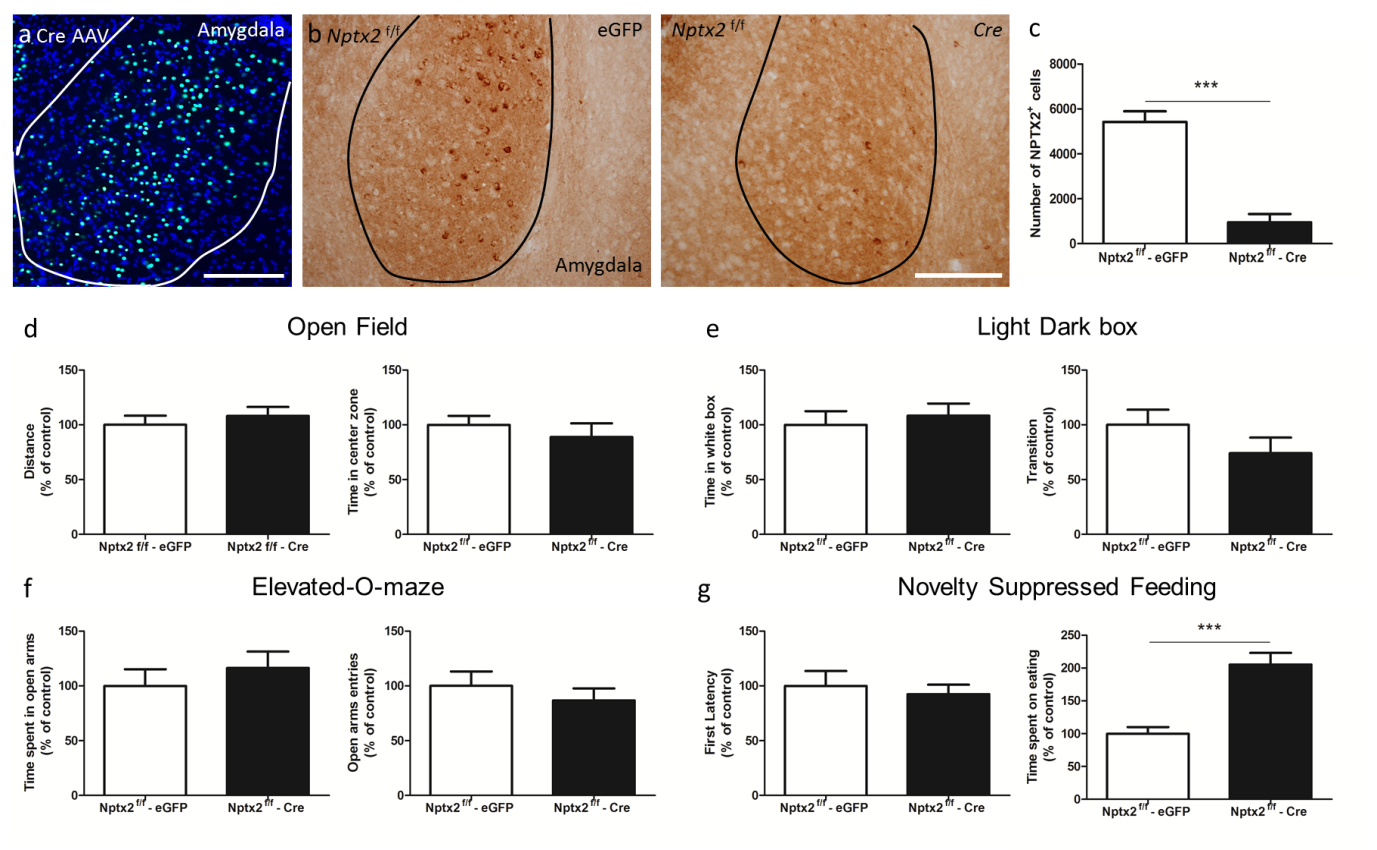
**

**Figure S4. Amygdala *Nptx2* knockdown by AAV-Cre injection does not alter anxiety**

(a-c) Viral vector expression (eGFP) and antibody staining of NPTX2 in amygdala. Immunohistochemistry confirmed there were significantly less NPTX2 positive cells in the amygdala after AAV-Cre injection. (d-g) Mice injected with AAV-*Cre* virus exhibited no significant difference in anxiety-like behavior as measured by open field, light dark box, and elevated-O-maze tests compared to controls. For the novelty suppressed feeding, amygdala *Nptx2* knockout mice spent more time eating but exhibited no difference in latency to approach food pellets (n = 8). Scale bar represents 200 µm. Values represent mean ± SEM. **p* < 0.05, ***p* < 0.01, ****p* < 0.001.

**
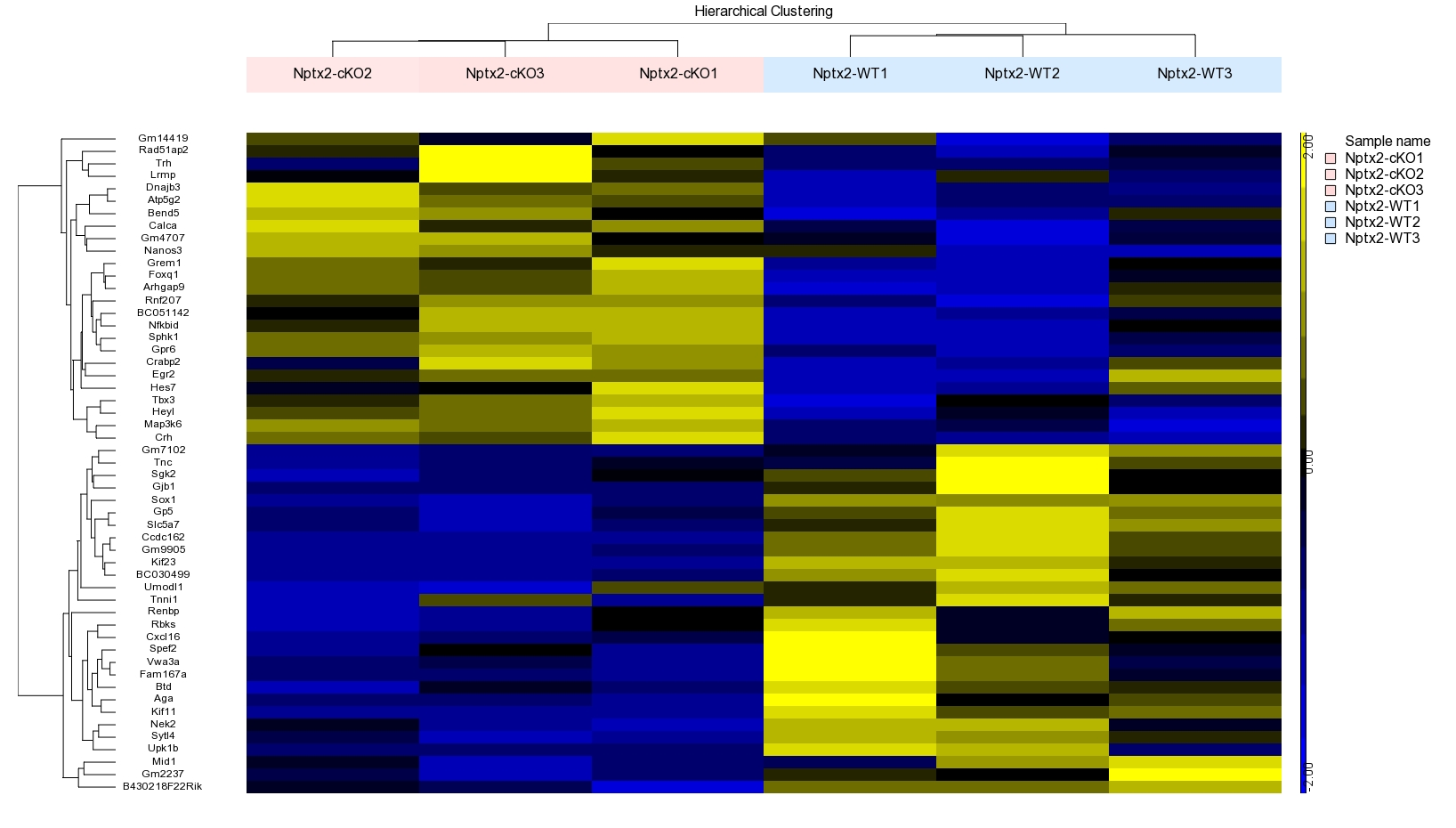
Figure S5. Heatmap and hierarchical clustering**

Heatmap and hierarchical clustering of 53 significantly up- and down- regulated genes in hippocampal tissue between *Nptx2* WT and cKO mice (n = 3 in each group, *p* < 0.05, fold change > 1.5 or < -1.5). Hierarchical clustering of the expression profiles was done using average linkage and Pearson correlations as a distance measure.

**
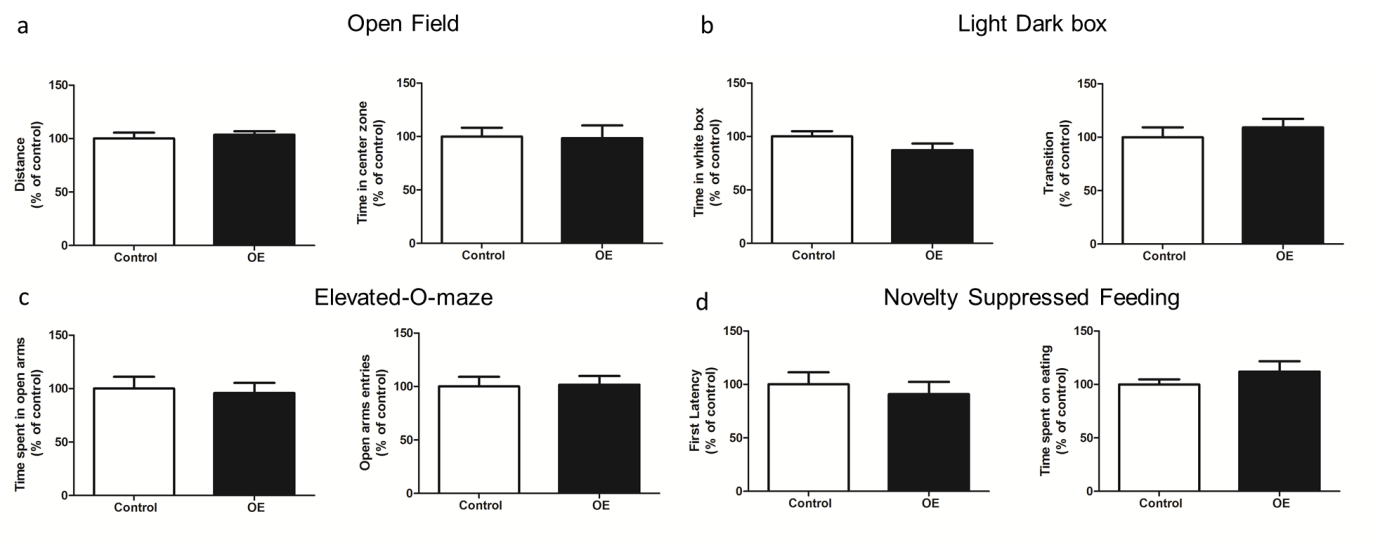
Figure S6. Overexpression of hippocampal *Nptx2* does not alter anxiety**

No significant difference in anxiety behaviors in C57BL/6 mice following intra-hippocampal injection of AAV-*Nptx2* overexpression viral vector (n = 10) in (a) Open field, (b) Light dark box, (c) Elevated-O-maze and (d) Novelty suppressed feeding tests. Values represent mean ± SEM. **p* < 0.05, ***p* < 0.01, ****p* < 0.001.
